# The crystal structure and biochemical characterization of Kif15: a bifunctional molecular motor involved in bipolar spindle formation and neuronal development

**DOI:** 10.1107/S1399004713028721

**Published:** 2013-12-24

**Authors:** Marta Klejnot, Aditi Falnikar, Venkatasubramanian Ulaganathan, Robert A. Cross, Peter W. Baas, Frank Kozielski

**Affiliations:** a The Beatson Institute for Cancer Research, Garscube Estate, Switchback Road, Glasgow G61 1BD, Scotland; b Department of Neurobiology and Anatomy, Drexel University College of Medicine, 2900 Queen Lane, Philadelphia, PA 19129, USA; c Warwick Medical School, University of Warwick, Coventry CV4 7AL, England; d School of Pharmacy, University College London, 29–39 Brunswick Square, London WC1N 1AX, England

**Keywords:** human kinesins, mitosis, bipolar spindle formation, Kif15, Eg5

## Abstract

Kinesins constitute a superfamily of microtubule-based motor proteins with important cellular functions ranging from intracellular transport to cell division. Some kinesin family members function during the mitotic phase of the eukaryotic cell cycle and are crucial for the successful progression of cell division. In the early stages of mitosis, during prometaphase, certain kinesins are required for the formation of the bipolar spindle, such as Eg5 and Kif15, which seem to possess partially overlapping functions. Because kinesins transform the chemical energy from ATP hydrolysis into mechanical work, inhibition of their function is a tractable approach for drug development. Drugs targeting Eg5 have shown promise as anticancer agents. Kif15 has recently come to the fore because it can substitute the functions of Eg5, and may itself have potential as a prospective drug target. Here, the initial biochemical, kinetic and structural characterization of Kif15 is reported and it is compared with the functionally related motor Eg5. Although Kif15 contains ADP in the catalytic site, its motor-domain structure was captured in the ‘ATP-like’ configuration, with the neck linker docked to the catalytic core. The interaction of Kif15 with microtubules was also investigated and structural differences between these two motors were elucidated which indicate profound differences in their mode of action, in agreement with current models of microtubule cross-linking and sliding.

## Abstract 2

The structural and biochemical study of Kif15 provides insight into this potential drug target and allows comparison with Eg5, a kinesin that partially shares the functions of Kif15.

## 1. Introduction   

Kinesins form a superfamily of proteins that play important roles in eukaryotic intracellular trafficking and cell division (Hirokawa *et al.*, 2009 ▶). The genomes of higher vertebrates contain as many as 45 genes coding for different kinesins. The majority of these molecular machines are implicated in intracellular transport, whereas a third of the superfamily members plays key roles in different stages of mitosis and cytokinesis (Wordeman, 2010 ▶). Some kinesins fulfill dual functions in both transport and cell division, such as Eg5 (KSP, Kif11, kinesin-5 family; Ferhat, Cook *et al.*, 1998 ▶), Kif15 (Hklp2, Xklp2, KRP180, KLP-18, kinesin-12 family; Buster *et al.*, 2003 ▶; Liu *et al.*, 2010 ▶), Kif2A (kinesin-13 family; Ganem & Compton, 2004 ▶; Zhu *et al.*, 2005 ▶), MKLP-1 (kinesin-6 family; Zhu *et al.*, 2005 ▶) and Kif4A/Kif4B (kinesin-4 family; Mazumdar *et al.*, 2004 ▶; Zhu *et al.*, 2005 ▶; Zhu & Jiang, 2005 ▶). A few kinesins are involved in the assembly and/or maintenance of the bipolar spindle (Tanenbaum & Medema, 2010 ▶), namely the two N-terminal plus-end-directed motors Eg5 (Blangy *et al.*, 1995 ▶) and Kif15, as well as the C-terminal minus-end-directed kinesin KifC1 (HSET, kinesin-13 family) and dynein, which belongs to another class of microtubule (MT)-based motor proteins (Vaisberg *et al.*, 1993 ▶).

Kif15 has been identified in a variety of eukaryotes, including *Xenopus* (Boleti *et al.*, 1996 ▶), human (Sueishi *et al.*, 2000 ▶), sea urchin (Rogers *et al.*, 2000 ▶), *Caenorhabditis elegans* (Segbert *et al.*, 2003 ▶) and rodents (Buster *et al.*, 2003 ▶). Although Kif15 and Eg5 appear to share some functional similarities during bipolar spindle formation, they seem to work through distinct mechanisms, adopting rather diverse quaternary structures (Vanneste *et al.*, 2009 ▶).

The involvement of Eg5 in the separation of the duplicated centrosomes (Blangy *et al.*, 1995 ▶) has made it a potential target for drug development in cancer chemotherapy, with several kinesin-5-specific inhibitors in multiple Phase I and II clinical trials (Huszar *et al.*, 2009 ▶; Rath & Kozielski, 2012 ▶). Interestingly, Kif15 overexpression has been shown to be able to restore functions of Eg5 under certain conditions, for example when Eg5 is depleted (Tanenbaum *et al.*, 2009 ▶). Subsequently, it has been hypothesized (although not yet proven) that tumours treated with Eg5-targeting drugs might acquire resistance to these inhibitors by simply up-regulating Kif15. This interesting hypothesis and the involvement of Kif15 in bipolar spindle assembly makes it a potential target for drug development in cancer chemotherapy, and the first inhibitor scaffolds targeting Kif15 have been reported in the patent literature (McDonald *et al.*, 2004 ▶).

Although the role of Kif15 in bipolar spindle formation has been investigated in a variety of organisms (Boleti *et al.*, 1996 ▶; Rogers *et al.*, 2000 ▶; Segbert *et al.*, 2003 ▶; Sueishi *et al.*, 2000 ▶) and has been compared with that of Eg5 in cellular assays, much less is known about its biochemical, mechanochemical and structural properties or its interactions with binding partners. The recombinant expression of human Kif15 for inhibitor screening, and the structure determination of its motor domain for structure-based design, would be highly desirable to facilitate the drug-development process. Here, we provide the initial biochemical characterization of human Kif15 and compare its properties with those of its functionally related partner Eg5. We reveal that Kif15, in contrast to Eg5, does not have a second nucleotide-independent MT-binding site in its C-terminal tail domain. Furthermore, we determine the crystal structure of the binary Kif15–ADP complex captured in the ‘ATP-like’ state adopted by the switch II cluster and neck-linker region.

## 2. Materials and methods   

### 2.1. Cloning, expression and purification of mammalian Kif15 proteins   

The *Homo sapiens* Kif15 motor domain and neck-linker region (named Kif15_19–375_ throughout this manuscript) was cloned, expressed and purified as described previously (Liu *et al.*, 2010 ▶). A longer construct including the first 19 residues that contains the cover strand (named Kif15_1–375_) was cloned by extending the Kif15_19–375_ construct by PCR with four forward primers (P1_F, 5′-CA AAT GGT CAG TCT AAC CAG CCG AGC AAC GAA G-3′; P2_F, 5′-GAG TTA CGC AGC GTG ACA AAT GGT CAG TCT AAC C-3′; P3_F, 5′-GGC TGC AAA ACT GAG TTA CGC AGC GTG-3′; P4_F, 5′-GAA **CC ATG G**CT CCT GGC TGC AAA ACT G-3′) and a single reverse primer (5′-GAT **CTC GAG** TTA ACC CTG GGT ATC TTC ATT CAC AAC C-3′). Kif15_1–375_ was expressed and purified as described for the shorter construct. The Kif15 tail construct from *Rattus norvegicus* (Kif15_1149–1388_) was cloned, expressed and purified as Kif15_19–375_.

### 2.2. Determination of protein concentrations   

Protein concentrations were determined either by using the Lambert–Beer law measured under native conditions [for the motor-domain constructs bound ADP was taken into account (2500 *M* ^−1^ cm^−1^)] or by applying the Bradford method. The extinction coefficients for each Kif15 construct were calculated (Kif15_1–375_, 17 670 *M* ^−1^ cm^−1^; Kif15_19–375_, 17 608 *M* ^−1^ cm^−1^; Kif15_1149–1388_, 10 033 *M* ^−1^ cm^−1^) using *ProtParam* from the Bioinformatics Resource Portal (Wilkins *et al.*, 1999 ▶).

### 2.3. Analytical gel filtration   

Analytical gel filtrations were performed on a Superose 12 10/300 GL column (GE Healthcare). Experiments were conducted at a flow rate of 0.5 ml min^−1^ (50 m*M* PIPES pH 6.8, 250 m*M* NaCl, 2 m*M* MgCl_2_, 1 m*M* DTT; all from Sigma) using an injection volume of 200 µl. Prior to running Kif15 expression constructs, the column was calibrated with proteins of known molecular mass (ribonuclease A, 13.7 kDa; carbonic anhydrase, 29 kDa; ovalbumin, 43 kDa; conalbumin, 75 kDa) and blue dextran. The *K*_av_ values were calculated for the calibration proteins [(*V*_e_ − *V*_0_)/(*V*_c_ − *V*_0_), where *V*_e_ is the elution volume, *V*_0_ is the void volume and *V*_c_ is the column volume] and were plotted against the log of the molecular masses of the standards. The molecular masses were calculated from the resulting equation.

### 2.4. Steady-state enzyme kinetics   

Steady-state basal and MT-stimulated activities were determined using the pyruvate kinase/lactate dehydrogenase coupled assay described previously (Hackney & Jiang, 2001 ▶). All measurements were carried out at 25°C using a 96-well Sunrise photometer (Tecan) in a final reaction volume of 100 µl at least in triplicate. Data were analysed using *Microsoft Excel* 2008 and *KaleidaGraph* 4.0 (Synergy Software). The salt dependence of the basal Kif15_19–375_ and Kif15_1–375_ ATPase activities was measured at NaCl concentrations from 0 to 275 m*M* in the presence of 0.59 µ*M* Kif15_19–375_ and 0.35 µ*M* Kif15_1–375_ and 1 m*M* Mg^2+^-ATP. To determine the basal ATPase activity of Kif15_19–375_ and Kif15_1–375_, their activities were examined at ATP concentrations from 0 to 2 m*M* in the presence of 0.35 µ*M* Kif15_19–375_ and 0.75 µ*M* Kif15_1–375_ as well as 75 m*M* NaCl for Kif15_19–375_ and 50 m*M* NaCl for Kif15_1–375_. The salt dependence of the MT-stimulated ATPase activity of Kif15_19–375_ and Kif15_1–375_ was determined by measuring their rates of activity at MT concentrations from 0 to 10 µ*M* in the presence of 0.04 µ*M* Kif15_19–375_ and 0.08 µ*M* Kif15_1–375_ (in the presence of 0, 50, 100 and 150 m*M* KCl for Kif15_19–375_ and in the presence of 0 and 50 m*M* KCl for Kif15_1–375_). Finally, the MT-stimulated ATPase activity for both Kif15 constructs was measured in the presence of increasing ATP concentrations from 0 to 2 m*M* in the presence of 0.04 µ*M* Kif15_19–375_ and 0.04 µ*M* Kif15_1–375_, 3 µ*M* MTs in the absence of salt.

### 2.5. Transient-state enzyme kinetics   

Slow Mant-ATP [2′/3′-*O*-(*N*-methylanthraniloyl)adenosine-5′-triphosphate] turnovers were performed by making manual additions to a 100 µl cuvette in a modified Cary Eclipse fluorescence spectrophotometer at 25°C. Reactions were started by adding the motor to a 5 µ*M* solution of Mant-ATP (Jena Bioscience) in BRB80 (80 m*M* PIPES–KOH pH 6.9, 1 m*M* MgCl_2_, 1 m*M* EGTA) to a final active-site concentration of 3.4 µ*M*. After the plateau of fluorescence had been reached, a 2.5 µl chase of unlabelled 100 m*M* Mg^2+^-ATP was added. Mant fluorescence was excited at 350 nm and emission was monitored at 450 nm. Stopped-flow experiments were performed at 25°C using a TgK SF-61DX2 stopped-flow spectrofluorimeter. Syringe 1 contained the Kif15_19–375_ or Kif15_1–375_ single head–Mant-ADP complex, which was formed by adding stock 5 m*M* Mant-ATP to 2 µ*M* Kif15 in BRB80 buffer with rapid mixing and incubating on ice for 30 min. Syringe 2 contained MTs at various concentrations plus 2 m*M* Mg^2+^-ATP chasing nucleotide. MTs for these experiments were assembled from pig brain tubulin in BRB80, stabilized using 20 µ*M* taxol and supplemented with 2 m*M* Mg^2+^-ATP immediately prior to use. Tubulin was dissolved in BRB80 without nucleotide or taxol. Mant fluorescence was excited at 350 nm and emission was monitored at 450 nm. Data were fitted to single exponentials to yield an apparent rate of Mant-ADP release (*k*_off_ in s^−1^) and an amplitude. Collected data for MT activation and tubulin activation of Mant-ADP release were fitted to rectangular hyperbolas using *KaleidaGraph*. Data points represent the averages of at least three pushes on at least two separate occasions.

### 2.6. Tubulin purification and polymerization into MTs   

Tubulin was purified from bovine brain by cycles of polymerization and depolymerization as described previously (Castoldi & Popov, 2003 ▶). Subsequently, tubulin was aliquoted at 18.5 mg ml^−1^, snap-frozen and stored in liquid nitrogen. MTs were prepared at 60 µ*M*. The MT concentration was calculated as the heterodimer concentration (110 kDa), assuming that all of the tubulin polymerized. Tubulin was gently mixed with pre-warmed G-PEM buffer (100 m*M* PIPES pH 6.9, 1 m*M* EDTA, 1 m*M* MgCl_2_, 1 m*M* GTP; all from Sigma) supplemented with 20 µ*M* taxol. Polymerization was carried out overnight at 37°C. For nucleotide binding and ATPase assays MTs were used in the taxol-polymerized form prepared without GTP.

### 2.7. Pelleting assays   

Pelleting assays were performed in 50 m*M* PIPES pH 6.9, 20 m*M* NaCl, 2 m*M* MgCl_2_, 1 m*M* DTT. Kif15_19–375_ (1–12 µ*M*) was gently mixed with taxol-polymerized MTs (5 µ*M*) and supplemented with buffer to a final volume of 40 µl. Binding assays were performed in the presence of 2 m*M* Mg^2+^-ATP, 2 m*M* AMP-PNP or 4 mU apyrase. Samples were incubated at room temperature for 10 min and centrifuged in a Beckman TL100 ultracentrifuge at 100 000*g* and 25°C for 20 min. Supernatants were removed from the pellets. The pellet was resuspended in Laemmli reducing sample buffer. Supernatants and pellet samples were run on gradient SDS–PAGE gels (4–12%). All measurements were performed in triplicate. Data were analyzed using *ImageJ* 143.u, *Microsoft Excel* 2008 and *KaleidaGraph* 4.0. To calculate the dissociation constant (*K*_d_), the Michaelis–Menten representation was used, with MT-bound Kif15_19–375_ ([MT·Kif15_19–375_]) plotted as a function of free Kif15_19–375_ ([Kif15_19–375_]),
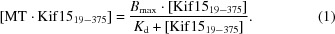

*B*_max_ corresponds to the maximal number of interacting sites.

### 2.8. Protein crystallization, data collection, processing and refinement   

Initial nanodrop screening was performed with purified Kif15_19–375_ at 10 mg ml^−1^ in the presence and absence of Mg^2+^-­ATP at 4 and 19°C. The protein crystallized at 19°C in the presence of nucleotide and 200 m*M* MgCl_2_, 25% PEG 3350, 100 m*M* Tris–HCl pH 8.5. Seeding further optimized the crystals, together with decreasing the protein concentration and the temperature. Improved crystals were immersed in cryoprotectant solution [20%(*w*/*v*) *meso*-erythritol, 240 m*M* MgCl_2_, 30% PEG 3350, 120 m*M* Tris–HCl pH 8.5] and flash-cooled in liquid nitrogen. Data were collected to a resolution of 2.7 Å at the European Synchrotron Radiation Facility (ESRF). The structure was solved by molecular replacement with *MOLREP* (Vagin & Teplyakov, 2010 ▶) using the CENP-E structure (PDB entry 1t5c; Garcia-Saez *et al.*, 2004 ▶) as a search model. The asymmetric unit contained three copies of Kif15_19–375_ with bound Mg^2+^-ADP in the catalytic site positioned with *REFMAC*5 (Murshudov *et al.*, 2011 ▶) by rigid-body and restrained refinement. The structure model was improved using *Coot* (Emsley & Cowtan, 2004 ▶) and further refined using *PHENIX* (Adams *et al.*, 2010 ▶) with noncrystallographic symmetry (NCS) restraints.

### 2.9. *In vitro* assay for neuronal migration   

We used a previously established *in vitro* culture system to study the migration of rat cerebellar granule neurons (Bix & Clark, 1998 ▶; Hirotsune *et al.*, 1998 ▶). Cerebella were isolated from 6–8-day-old rat pups, triturated to give a single-cell suspension and then transfected with either control or Kif15 siRNA and control EGFP plasmid using an Amaxa electroporator and plated on polylysine-coated plastic dishes in serum-containing medium. The next day, the cells were released by treatment with trypsin and were allowed to stand in a solution overnight after removing the trypsin. During this period the cells formed aggregates, which were plated on glass cover slips coated with laminin in serum-free medium to stimulate migration. For time-lapse imaging, the cover slips were placed in an imaging station consisting of a Zeiss environ­mental chamber and a Zeiss Observer microscope. Serial images were captured every 2 min for a period of 4 h. To quantify cell movement, the total displacement exhibited by the cell body was measured using the *AxioVision* software and was divided by the time taken for the displacement.

## 3. Results and discussion   

### 3.1. Biophysical characterization of mammalian Kif15 motor and tail domains   

**Figure 1:**
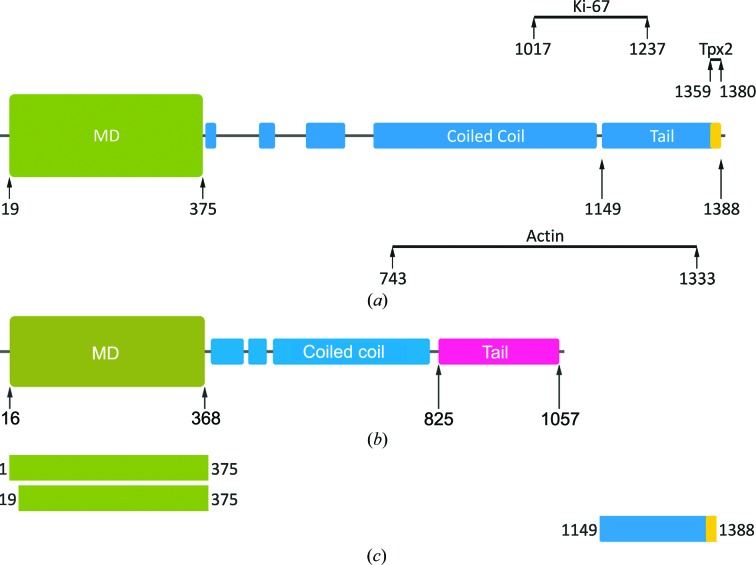
Schematic representation of human Kif15 and Eg5. (*a*) The Kif15 motor domain shown in green is followed by a discontinuous α-helical region predicted to form a coiled-coil stalk domain (coloured blue). The C-terminal leucine-zipper motif is shaded in yellow. The putative interacting regions for actin (residues 743–1333), Ki-67 (residues 1017–1237) and TPX2 (residues 1359–1380) in the C-­terminal half of Kif15 are also indicated. (*b*) The Eg5 motor domain shown in green is followed by a discontinuous α-helical region forming a coiled-coil domain (coloured blue). The C-terminal globular tail is marked in pink. (*c*) Bar diagrams of the Kif15 constructs used in this study.

Human Kif15 is a protein of 1388 residues (Fig. 1 ▶ *a*) with an N-terminal motor domain (19–375) followed by a long α-­helical rod-shaped stalk predicted to form an interrupted coiled coil. A bar diagram of Eg5 is presented in Fig. 1 ▶(*b*). Kif15 does not contain the conventional C-terminal globular domain typical of many other kinesins (*e.g.* Eg5); it possesses a C-terminal leucine zipper, a common dimerization motif (residues 1359–1380). The C-terminal region has also been shown to contain binding sites for the forkhead-associated (FHA) domain of Ki-67 (Sueishi *et al.*, 2000 ▶; Vanneste *et al.*, 2009 ▶) and for the nuclear protein TPX2 (targeting protein for Xklp2; Tanenbaum *et al.*, 2009 ▶) (Fig. 1 ▶ *a*). The internal region of Kif15 contains a putative myosin tail homology domain, which has been shown to enable Kif15 to co-localize with actin (Buster *et al.*, 2003 ▶).

We subcloned, expressed and purified the Kif15 motor (Kif15_19–375_, coding for residues 19–357) and Kif15_1–375_ including the first 18 residues that are thought to include the cover strand, a short nine-residue N-­terminal region shown to interact with the C-terminal neck-linker region. These two regions have been shown to fold into the so-called neck-cover bundle by forming a small β-sheet and represent a force-generating element in several members of the kinesin superfamily (Hwang *et al.*, 2008 ▶; Khalil *et al.*, 2008 ▶). Finally, we cloned the C-terminal tail construct (named Kif15_1149–1388_; Fig. 1 ▶ *c*) using *Escherichia coli* codon-optimized cDNA. The identity of the purified proteins was verified by mass-spectrometric fingerprint analysis, with a sequence coverage of 65% for Kif15_19–­375_, 81% for Kif15_1–375_ and 61% for Kif15_1149–1388_. The molecular masses of Kif15_19–375_ and Kif15_1–375_ determined by gel filtration were 37 and 39 kDa close to their theoretical masses of 37 and 39 kDa, respectively, indicating that the Kif15 motor domain and the Kif15 motor including the cover-strand region are monomeric, in agreement with other kinesins. The gel-filtration profile for Kif15_1149–1388_ was heterogeneous, indicating possible assembly into higher oligomers and/or partial aggregation.

### 3.2. Crystal structure of the binary Kif15_19–375_–Mg^2+^-ADP complex   

The structure of the Kif15 motor domain was solved by molecular replacement and refined to a resolution of 2.7 Å. Data-collection and refinement statistics are shown in Table 1 ▶. Kif15_19–375_ crystallized in space group *P*3_2_21 with three molecules in the asymmetric unit. The crystals have a Matthews parameter of 2.4 Å^3^ Da^−1^ and a solvent content of 49.1%. The r.m.s. deviations from ideal geometry were 0.013 Å for bond lengths and 1.60° for bond angles for the refined structure. The final model contains residues 24–375 for molecules *A* and *B* and residues 24–372 for molecule *C*. Owing to missing or non-interpretable electron density, several smaller loops could not be built and are absent from the model. Side chains for which no density was observed were deleted from the C^β^ position onwards. The Ramachandran plot shows that 94.3% of the residues are in favoured regions, 5.5% are in additionally allowed regions and 0.2% are outliers. Since molecule *A* presented better defined electron density and a more complete model than molecules *B* and *C*, we subsequently use it for further descriptions of the structure.

**Figure 2:**
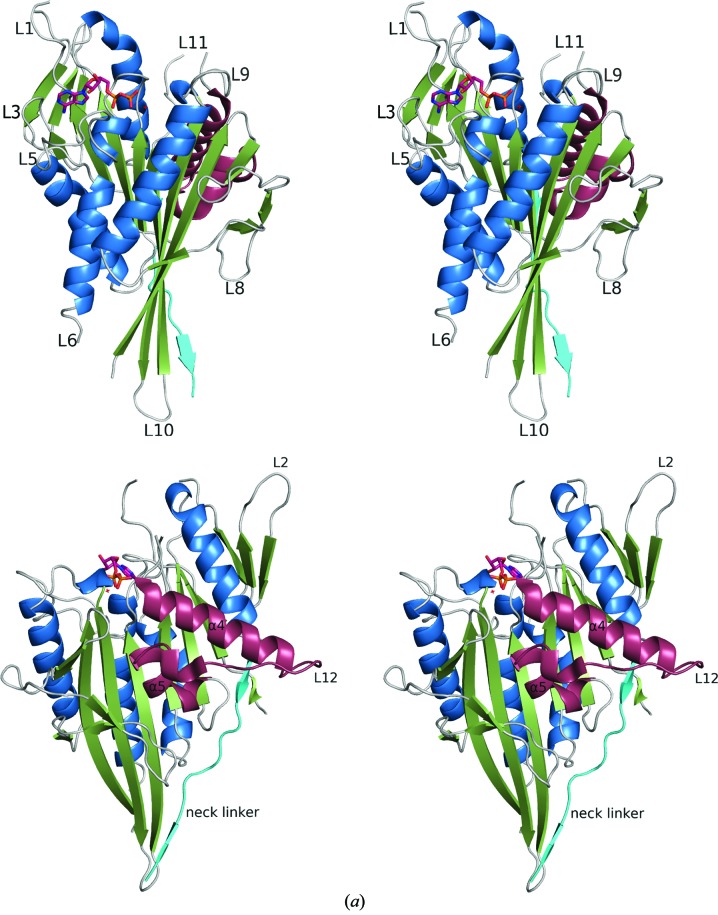

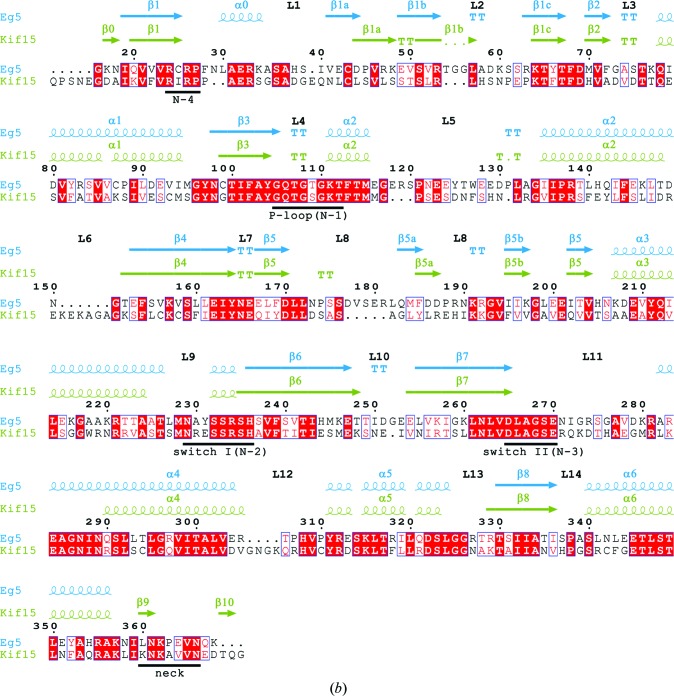
Overall structure of the binary Kif15_19–375_–Mg^2+^-ADP complex and comparison of the sequence/secondary structure of the Kif15 and Eg5 motor domains. (*a*) Stereo plots of the front and back view of the human Kif15 motor domain. α-Helices are coloured blue, β-strands green and loops/turns grey. The switch II cluster (α4–L12–α5) is highlighted in claret and the neck linker following the C-terminal helix α6 is shown in cyan. Mg^2+^-ADP is shown as a ball-and-stick model. (*b*) Structural and sequence alignment of the Eg5 (PDB entry 3hqd; Parke *et al.*, 2009 ▶) and Kif15 motor domains. Residue 16 of Eg5 is aligned with residue 24 of Kif15. Identical residues are coloured white on a red background and similar residues are shaded in red. The position of the ATP-binding pocket (N1–N4), the switch I and II regions and the position of the neck-linker regions are underlined in black.

The Kif15 motor domain shows the typical kinesin fold with an eight-stranded β-sheet surrounded by three major α-helices on each side (Fig. 2 ▶ *a*). The crystallized Kif15 construct contains the neck linker, which aligns with the catalytic core, forming a small β-sheet. Although Mg^2+^-ADP is bound in the catalytic site, Kif15 is in the so-called ‘ATP-like’ conformation. The switch II cluster (helix α4–loop L12–helix α5) is in the so-called ‘upward’ position, allowing the neck linker to dock to the motor domain (Fig. 2 ▶ *a*). We subsequently compared the structure of the Kif15 motor with that of the functionally related Eg5 (Blangy *et al.*, 1995 ▶; Turner *et al.*, 2001 ▶), using the AMP-PNP-bound complex representing the ‘ATP-like’ state of Eg5 (Parke *et al.*, 2009 ▶). Sequence alignment of Kif15 and Eg5 shows their high similarity inside their motor domains, with 41.8% identical, 15.5% strongly similar and 12.8% weakly similar residues, which is also reflected by their structures (Fig. 2 ▶ *b*). Outside their motor domains the primary sequence is at best only weakly conserved, with 20.3% identical, 20.2% strongly similar and 9.8% weakly similar residues. Kif15 has a deletion of two amino acids and a single insertion in the loop L1 region. In the case of Eg5 loop L1 is interrupted by helix α0, while in Kif15 loop L1 is longer. Loop L1 is part of the L1–L3–L5 cluster that together with loop L9 mediates the entrance of the nucleotide (Song *et al.*, 2001 ▶). In contrast to Eg5, three residues are missing in the Kif15 loop L2 region. Four residues are missing in loop L5, five in loop L8 (loop L8 is implicated in MT-stimulated ADP release; Fourniol & Moores, 2010 ▶) and a single residue is missing in the surface loop L10. There is a five-residue insertion in loop L6 of Kif15 which is believed to influence the nucleotide affinity (Kollmar & Glöckner, 2003 ▶). In Eg5, loop L5 is known to be important for inhibitor binding, and differences in the length of this motif in Kif15 (in Kif15 this loop is shorter) explain why Eg5-targeting inhibitors do not inhibit Kif15 (Liu *et al.*, 2010 ▶). In addition, several other key residues involved in inhibitor binding in Eg5, such as Glu116, Arg119, Trp127 and Ala216, are not present in Kif15. Loop L11 is not visible in any of the three molecules in the asymmetric unit.

### 3.3. Steady-state ATPase activity   

**Figure 3:**
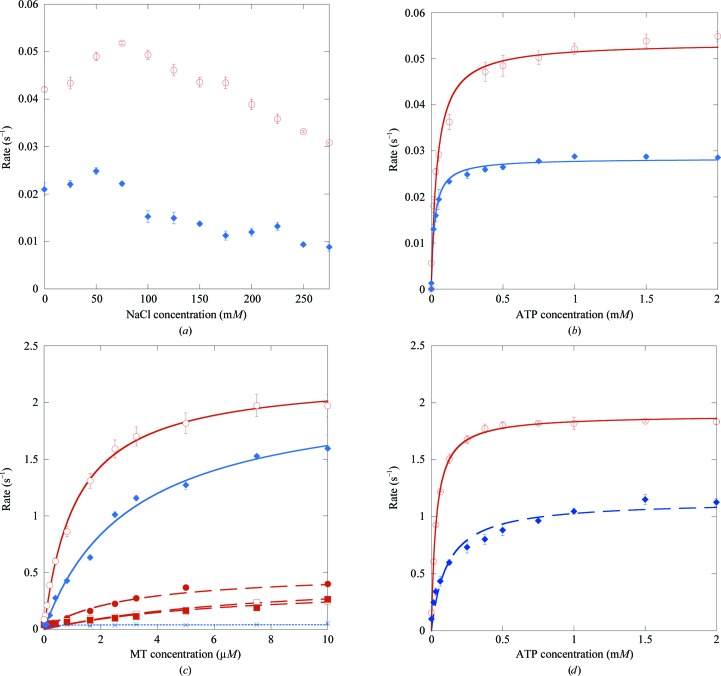
Characterization of the basal and MT-stimulated ATPase activities of Kif15_19–375_ and Kif15_1–375_. (*a*) Influence of the NaCl concentration on the basal ATPase activity of Kif15_19–375_ (red) and Kif15_1–375_ (blue) in the presence of 1 m*M* ATP. (*b*) Optimization of the basal ATPase activity in the presence of increasing ATP concentrations measured at 75 m*M* NaCl for Kif15_19–375_ (red) and 50 m*M* NaCl for Kif15_1–375_ (blue). (*c*) Salt dependence of the MT-stimulated Kif15_19–375_ ATPase activity (red) in the absence (circles) and in the presence of 50 m*M* (filled circles), 100 m*M* (squares) and 150 m*M* (filled squares) KCl and salt dependence of the MT-stimulated Kif15_1–375_ ATPase activity (blue) in the absence (filled diamonds) and the presence (squares) of 50 m*M* KCl. Data were measured at increasing MT concentrations ranging from 0 to 10 µ*M* in the presence of 1 m*M* ATP. (*d*) Optimization of the ATP concentration for the MT-stimulated ATPase activity of Kif15_19–375_ (red) and Kif15_1–375_ (blue) in the presence of increasing ATP concentrations, measured at 3 µ*M* MTs, in the absence of salt.

We subsequently measured the kinetic parameters for Kif15_19–375_ and Kif15_1–375_ (Fig. 3 ▶ and Table 2 ▶). To determine the optimal salt concentration, the rates of basal ATPase activity were measured on varying the NaCl concentration from 0 to 275 m*M*. The highest basal activity was measured at 75 m*M* NaCl for Kif15_19–375_ and at 50 m*M* for Kif15_1–375_ (Fig. 3 ▶ *a*). The differences in activity were rather minor and only decreased slowly at higher salt concentrations. The basal ATPase activity was measured using ATP concentrations from 0 to 2 m*M*. The *k*_cat_ for the basal ATPase activity of Kif15_19–375_ was 0.054 ± 0.001 s^−1^, with a *K*_m,ATP_ of 40.5 ± 5.4 µ*M* (Fig. 3 ▶ *b*, red data points). The *k*_cat_ for the basal ATPase activity of Kif15_1–375_ was 0.028 ± 0.0004 s^−1^, about twofold lower, with a *K*_m,ATP_ of 23.0 ± 1.9 µ*M* (Fig. 3 ▶ *b*, blue data points), an almost twofold difference but still similar to the construct without the cover strand. Subsequently, the salt dependence of the MT-stimulated ATPase activity of Kif15_19–375_ and Kif15_1–375_ was determined by measuring the rates at MT concentrations from 0 to 10 µ*M* in the presence of 0, 50, 100 or 150 m*M* KCl for Kif15_19–375_ and of 0 or 50 m*M* KCl for Kif15_1–375_. With increasing salt concentrations the MT-stimulated ATPase activity for both Kif15 constructs decreased significantly (Fig. 3 ▶ *c*). The *k*_cat_ for the MT-stimulated ATPase activity was 2.3 ± 0.1 s^−1^ with a *K*_0.5,MT_ of 1.1 ± 0.1 µ*M* for Kif15_19–375_, and *k*_cat_ was 2.1 ± 0.1 s^−1^ with a *K*_0.5,MT_ of 3.1 ± 0.3 µ*M* for Kif15_1–375_, indicating that there are no differences in the *k*_cat_ values between these two Kif15 constructs. Finally, the MT-­stimulated ATPase activity in the presence of increasing ATP concentrations (from 0 to 2 m*M*) was determined (Fig. 3 ▶ *d*). The *K*_m,ATP_ values were 33 ± 3 µ*M* for Kif15_19–375_ and 109 ± 20 µ*M* for Kif15_1–375_. With the exception of a threefold difference in the *K*_0.5,MT_ and *K*_m,ATP_ values there was no significant difference between these two constructs, indicating that the presence or absence of the cover strand does not significantly change the ATPase characteristics.

### 3.4. Transient-state ATPase activity   

**Figure 4:**
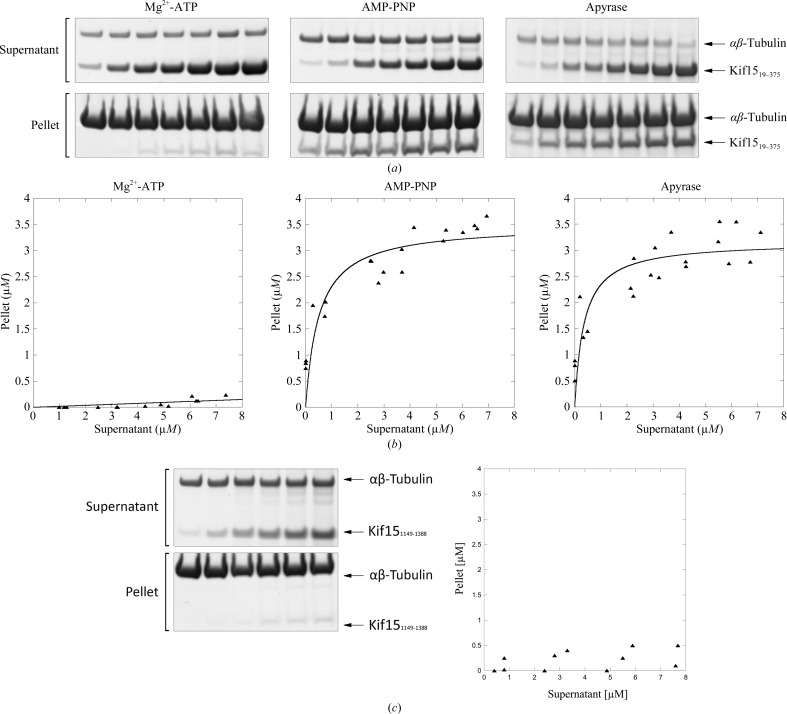
MT pelleting assays in the presence of Kif15 motor or tail domains. (*a*) MT pelleting assays of the Kif15 motor domain in the presence of various nucleotides. Increasing amounts of Kif15_19–375_ (1–12 µ*M*) were incubated with MTs (5 µ*M*) in the presence of 2 m*M* Mg^2+^-ATP, 2 m*M* AMP-PNP or 4 mU apyrase. Samples of supernatants and pellets were analysed by SDS–PAGE. (*b*) MT binding of Kif15_19–375_ in the presence of 2 m*M* Mg^2+^-ATP, 2 m*M* AMP-PNP or 4 mU apyrase. The plotted data relate to the amounts (µ*M*) of Kif15_19–375_ recovered from supernatant and pellet (pelleted with MTs) fractions of reactions run in the presence of various nucleotides. Data were obtained by analysing the SDS–PAGE (*ImageJ* 143.u) presented in (*a*). (*c*) MT pelleting assays of the Kif15 tail domain. Increasing amounts of Kif15_1149–1388_ (1–10 µ*M*) were incubated with MTs (5 µ*M*). Samples of supernatants and pellets were analysed by SDS–PAGE. The plotted data relate to the amounts (µ*M*) of Kif15_1149–1388_ recovered from supernatant and pellet (pelleted with MTs) fractions. Data were obtained by analysing the SDS–PAGE (*ImageJ* 143.u) presented on the left side of the figure.

Mant-ATP is a fluorescent analogue of ATP that for some kinesins gives a fluorescent enhancement on binding at the motor active site. Initial slow transient-state turnover assays of Mant-ATP were performed by making manual additions to a fluorimeter cuvette (Supplementary Fig. S1*a* 1). As can be seen in our crystal structure, Kif15, like other kinesins, purifies with Mg^2+^-ADP in its active site (Fig. 2 ▶ *a*). Addition of an excess of Mg^2+^-Mant-ATP initiates replacement of this active-site Mg^2+^-­ADP with Mant-ADP at a rate limited by the basal rate constant of Mg^2+^-ADP release from the Kif15 motor domain. We estimate this rate constant to be 0.017 s^−1^. Once a plateau of enhanced fluorescence is reached, addition of a ‘chase’ of nonfluorescent Mg^2+^-ATP (Supplementary Fig. S1*a*) initiates the opposite reaction, in which active-site Mg^2+^-Mant-ADP is replaced with chasing Mg^2+^-ADP. At 0.005 s^−1^, the rate constant for Mant-ADP release is approximately threefold slower than that for Mg^2+^-ADP release, indicating that Mant-ADP binds slightly tighter than Mg^2+^-ADP but is nonetheless a reasonably faithful surrogate and reporter for Mg^2+^-ADP release. Preliminary stopped-flow assays were performed to examine the MT activation of Mant-ADP release (Supplementary Fig. S1*b*). Kif15 was pre-incubated on ice to load the active site with Mant-ATP. The complex was then rapidly warmed to 25°C, loaded into the stopped flow and rapidly mixed with various concentrations of MTs in the presence of an excess of unlabelled chasing MgATP. The stopped-flow data are preliminary, but nonetheless show that MTs activate MgADP release from Kif15 motor domains ∼1000-fold. The apparent affinity for MTs in the presence of ATP was consistent with steady-state assays (Fig. 3 ▶ *c*) and MT pull-down experiments (Figs. 4 ▶ *a* and 4 ▶ *b*).

Comparison of the steady-state ATPase kinetic parameters of Eg5 and Kif15 show that they exhibit optimal *in vitro* activity at higher salt concentrations (Eg5, 150 m*M*; Kif15, 50–75 m*M*). The two motors have similar *K*_m_ and *k*_cat_ values for their basal activity, but the MT-activated ATPase activity is higher in the case of Eg5 than of Kif15. The affinities of the motor domains for MTs vary similarly according to the type of bound nucleotide (Lockhart & Cross, 1996 ▶), but steady-state, transient-state and direct binding measurements all indicate that the *K*_0.5,MT_ is considerably higher in the case of Kif15.

### 3.5. Kif15 contains a nucleotide-dependent MT-binding site in its motor domain, but not in its tail domain   

We then determined the affinity of Kif15_19–375_ for MTs in the presence of Mg^2+^-ATP, AMP-PNP and apyrase (thus establishing the ‘nucleotide-free’ state) using the MT pelleting assay. The results for the Kif15 motor domain are shown in Fig. 4 ▶ and the *K*_d_ values as well as the stoichiometry of the Kif15–MT complex are summarized in Table 3 ▶. In the presence of the slowly hydrolysable ATP analogue AMP-PNP or apyrase, Kif15_19–375_ shows comparable affinities for MTs, with *K*_d_ values of 0.5 ± 0.2 and 0.4 ± 0.2 µ*M*, respectively, and a stoichiometry close to one (1.1 and 1.3, respectively), indicating that one Kif15 motor domain binds to one αβ-tubulin heterodimer, in agreement with the results reported for other kinesins. Under the conditions tested the Kif15 motor domain shows no affinity for MTs in the presence of Mg^2+^-ATP (in the presence of MTs ATP is rapidly hydrolysed to ADP, which is known to possess low affinity for MTs). In conclusion, human Kif15 contains a nucleotide-dependent MT-binding region in its motor domain.

We then investigated whether or not Kif15 contains a nucleotide-independent MT-binding region in its tail domain (Fig. 4 ▶ *c*). A second MT-binding site has been identified in a variety of kinesin tail domains (Liao *et al.*, 1994 ▶; Germani *et al.*, 2000 ▶; Kuriyama *et al.*, 1994 ▶; Echard *et al.*, 1998 ▶; Chandra *et al.*, 1993 ▶; Karabay & Walker, 1999 ▶; Meluh & Rose, 1990 ▶; Narasimhulu & Reddy, 1998 ▶; Jiang *et al.*, 2007 ▶) and has been shown in some cases to be physiologically important for their biological functions. In our hands Kif15_1149–1388_ does not bind to MTs in pelleting assays, indicating that the tail does not contain an MT-binding side. This in contrast to human Eg5, which has recently been shown to contain a nucleotide-independent MT-binding region in its tail which is implicated in MT cross-linking and sliding (Weinger *et al.*, 2011 ▶).

### 3.6. Functional comparison of Kif15 and Eg5 in a non-mitosis scenario   

**Figure 5:**
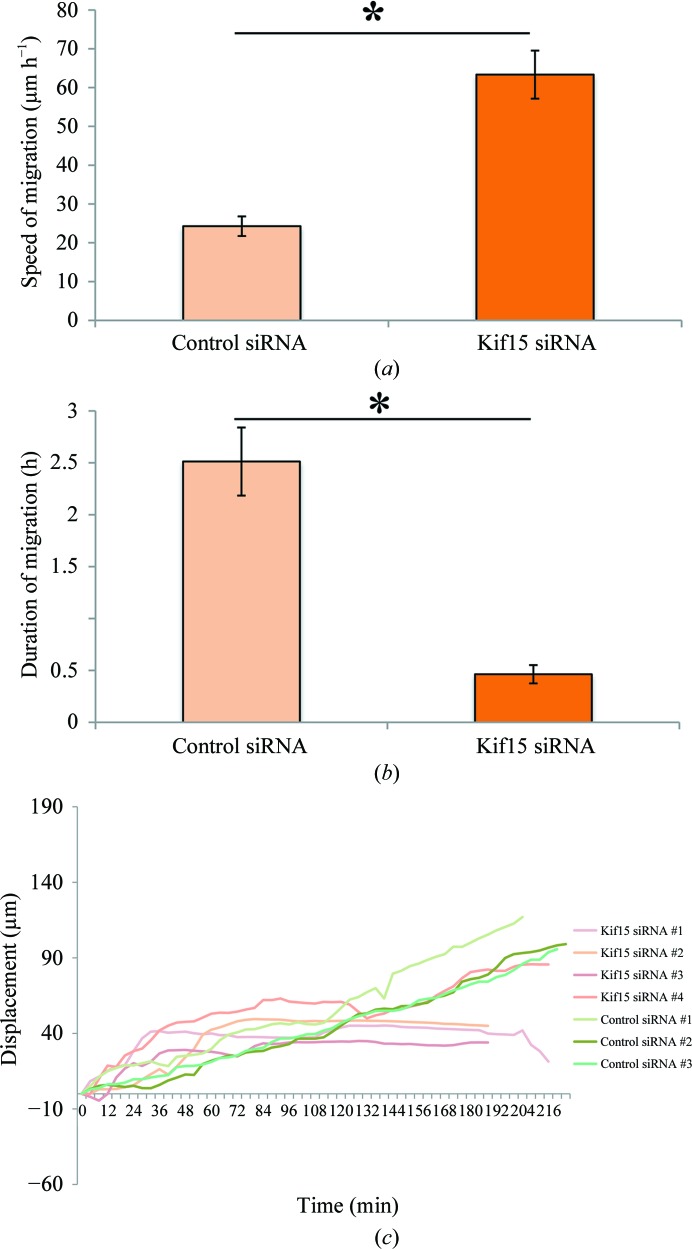
Depletion of Kif15 results in faster moving migratory neurons, which migrate less consistently. (*a*) Quantification of average speed of migration for control siRNA and Kif15 siRNA-treated cells (*n* = 11 and *n* = 12 cells, respectively). (*b*) Quantification of the time period for control siRNA and Kif15 siRNA-treated neurons, during which they exhibited consistent forward movement. (*c*) Tracing of movements of individual cell bodies.

Given that the mitotic spindle is a rather unique MT apparatus, we wished to investigate whether these two different motors could accomplish similar functions in a non-mitosis scenario. It has previously been reported that Eg5 as well as Kif15 are both enriched in the populations of neurons undergoing migration in the developing brain (Ferhat, Kuriyama *et al.*, 1998 ▶; Buster *et al.*, 2003 ▶). A role for Eg5 in regulating neuronal migration has already been documented such that depletion of Eg5 results in neurons that migrate faster (Falnikar *et al.*, 2011 ▶). Here, we tested whether Kif15 plays a similar role by depleting it from migrating cerebellar granule neurons using siRNA followed by time-lapse imaging. We found that neurons depleted of Kif15 typically migrated faster but less consistently, such that after a period of vigorous forward movement a cell either remained stationary or underwent a temporary stationary phase before restarting the next phase of forward movement. Quantification showed that the average displacement exhibited by neurons transfected with control siRNA was 24.3 ± 2.5 µm h^−1^, whereas that for neurons transfected with Kif15 siRNA was 63.3 ± 6.1 µm h^−1^ (*p* < 0.0001; see Fig. 5 ▶ *a*). The average time period during which neurons exhibited consistent forward movement was 2.5 ± 0.32 h for the control siRNA group, whereas that for the Kif15 siRNA group was 0.46 ± 0.09 h (*p* < 0.0001; see Fig. 5 ▶ *b*). This phenotype differs from the Eg5-depletion phenotype in terms of the consistency of migration. For tracings of the movements of individual cell bodies, see Fig. 5 ▶(*c*). These observations are reminiscent of previous studies on axonal growth and guidance, in which depleting each of the two motors produced similar but not identical phenotypes (Liu *et al.*, 2010 ▶).

### 3.7. Kif15 and Eg5 in mitosis   

**Figure 6:**
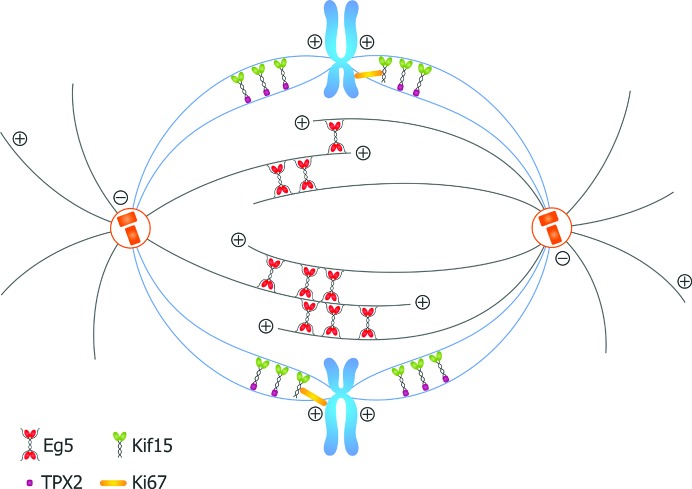
Comparison of current models for Eg5 and Kif15 MT crosslinking and function. Under physiological conditions homotetrameric Eg5 (blue) interacts with antiparallel MTs through two distinct binding sites in its motor and tail domains (two in the motor and two in the tail for each MT it crosslinks), and slides them apart (Weinger *et al.*, 2011 ▶). Dimeric Kif15 (green) works predominantly on K-fibres (Sturgill & Ohi, 2013 ▶). It forms a complex with TPX2 (pink) *via* its tail domain to bind statically to one MT while moving along a second (Tanenbaum *et al.*, 2009 ▶). It can also interacts with chromosomes via its interaction wit FHA domain of Ki-67 (Sueishi *et al.*, 2000 ▶). The stoichiometry of the Kif15–Ki-67 and the Kif15–TPX2 complex is unknown.

The role of Kif15 during bipolar spindle formation in early prometaphase reveals that it may – under certain conditions – be a functional homologue of Eg5. Although their mechanism of action is clearly different (Fig. 6 ▶), both proteins are able to work redundantly to fulfill their similar roles. Homotetrameric Eg5 performs its function because of its unique quaternary structure that allows it to cross-link antiparallel MTs and, with its plus-ended directed motility, to slide them apart to form the bipolar spindle. It has recently been suggested that the mechanistics of Eg5 function are even more complex, with additional MT binding sites located in the tail domains (Weinger *et al.*, 2011 ▶). In contrast, Kif15 works as a dimer (Wittmann *et al.*, 1998 ▶) by crosslinking parallel kinetochore-MTs (Sturgill & Ohi, 2013 ▶) and we demonstrated that it does not contain an MT-binding site within its tail domain. The function of the MT linker is most likely carried out by TPX2 through the C-terminal leucine-zipper region of Kif15 (Wittmann *et al.*, 2000 ▶). Owing to their similar functions, Kif15 may be able to take over the role of Eg5 following anti-Eg5 chemotherapy by simply up-regulating protein-expression levels, at least in cell culture (Tanenbaum *et al.*, 2009 ▶). Such an interchange ability of the two motors, albeit not perfect, is supported by our observations on neurons, which are non-mitotic, suggesting a broadly applicable theme by which two structurally different motors can be made to work in very similar ways. If the scenario of a possible resistance mechanism were true for certain tumours, co-inhibition of both of motors, Eg5 and Kif15, could be a vital therapeutic approach.

This paper reports the crystal structure of human Kif15, which will be a valuable asset for structure-guided design. The comparison of both motors revealed that despite overall functional similarity, Kif15 and Eg5 have important differences, allowing Kif15 to be impervious to Eg5-targeting drugs.

## Supplementary Material

PDB reference: Kif15_19–375_, 4bn2

Supporting Information. DOI: 10.1107/S1399004713028721/dw5063sup1.pdf

## Figures and Tables

**Table 1:** Data-collection and refinement statistics for the *H. sapiens* Kif15_19–375_–Mg^2+^-ADP binary complex Values in parentheses are for the highest resolution shell.

PDB entry	4bn2
Data-collection statistics	
Beamline	ID23-1, ESRF
Detector	ADSC Q315R
Resolution range	30–2.7
Space group	*P*3_2_21
Unit-cell parameters (Å, °)	*a* = *b* = 90.2, *c* = 251.4, α = β = 90, γ = 120
Completeness (%)	89.7 (86.5)
*R*_merge_ (%)	9.8 (37.8)
Multiplicity	3.7 (3.5)
Mean *I*/σ(*I*)	8.6 (3.1)
Total No. of reflections	108223
No. of unique reflections	29592
No. of copies per asymmetric unit	3
Refinement statistics	
*R*_work_/*R*_free_ (%)	21.2/26.3
No. of Mg^2+^-ADP molecules	3
No. of water molecules	156
R.m.s.d. from ideal geometry	
Bond lengths (Å)	0.013
Bond angles (°)	1.60

**Table 2:** Kinetic parameters determined for Kif15_19–375_ and Kif15_1–375_

	Basal ATPase activity		MT-stimulated ATPase activity		
	*k*_cat_ (s^−1^)	*K*_m,ATP_ (µ*M*)	*k*_cat_ (s^−1^) ([KCl], m*M*)	*K*_0.5,MT_ (µ*M*)	*K*_m,ATP_ (µ*M*)
Kif15_19–375_	0.054 ± 0.001	40.5 ± 5.4	2.3 ± 0.1 (0)	1.1 ± 0.1	33 ± 3
			0.5 ± 0.1 (50)		
			0.5 ± 0.1 (100)		
			0.4 ± 0.1 (150)		
Kif15_1–375_	0.028 ± 0.0004	23.0 ± 1.9	2.1 ± 0.1 (0)	3.1 ± 0.3	109 ± 20
			0.05 ± 0.01 (50)		

**Table 3:** Binding of the Kif15 motor domain to MTs in two different nucleotide states Dissociation constants (*K*_d_) and the stoichiometry of binding of Kif15_19–375_ to MTs measured in the presence of 20 m*M* NaCl. Values for Mg^2+^-ATP were not determined.

	AMP-PNP	Apyrase
*K*_d_ (µ*M*)	0.5 ± 0.2	0.4 ± 0.2
Stoichiometry	1:1.1	1:1.3

## Footnotes

1 Supporting information has been deposited in the IUCr electronic archive (Reference: DW5063).
