# Supplementary material for: The crystal structure and biochemical characterization of Kif15: a bifunctional molecular motor involved in bipolar spindle formation and neuronal development
Source: Acta Crystallogr D Biol Crystallogr. 2013 Dec 24;70(Pt 1):123–33. doi: 10.1107/S1399004713028721 (PMC3919264; doi:10.1107/S1399004713028721)
Supplement: Supplementary file 1 [file d-70-00123-sup1.pdf]

Supporting Information

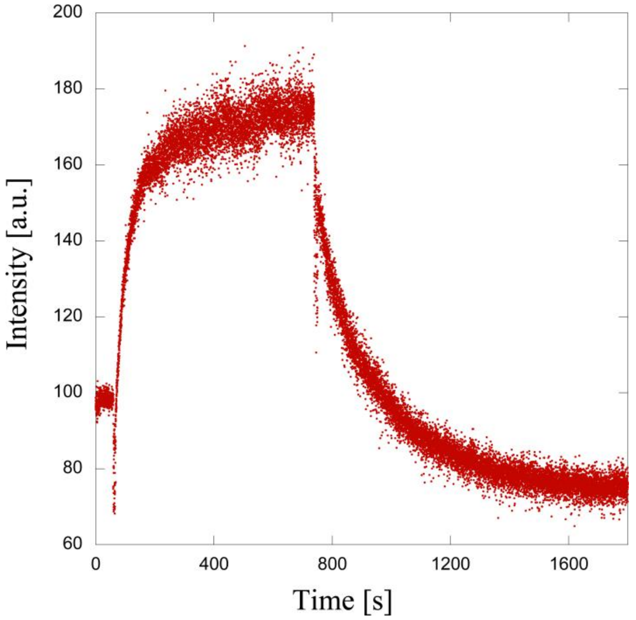

(a)

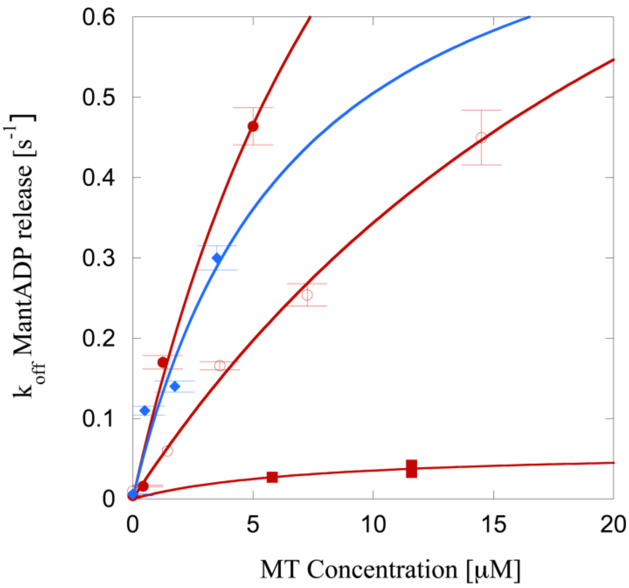

(b)

Supporting Figure S1 Transient state ATPase activity of of $Kif15_{19\text{-}375}$ and $Kif15_{1\text{-}375}$. **a)** Transient state ATP turnover. Addition of $Kif15_{19\text{-}375}$ to a solution of MantATP at t = ~50 s initiates a fluorescence enhancement as the MantATP binds. Addition of chasing Mg^{2+}ATP on the plateau of enhanced fluorescence at t = ~750 s initiates a decay corresponding to the release of MantADP. Exponential fits to the rising and decay phases yield rate constants k_{on} = 0.017 s^{-1} for basal Mg^{2+}ADP release and k_{off} = 0.005 s^{-1} for Mg^{2+}MantADP release. **b)** Stopped flow data for MT activation of $Kif15_{19\text{-}375}$ MantADP release in BRB20 (**o**) and BRB80 for $Kif15_{19\text{-}375}$ (•) and $Kif15_{1\text{-}375}$ (◆). Data for both $Kif15_{1\text{-}375}$ and $Kif15_{19\text{-}375}$ are fitted

to rectangular hyperbolae to yield estimates for the maximum rate of MT-activated MantADP release and for $K_{0.5,\ MT,}$ the apparent Michaelis constant for MT activation. For $Kif15_{19\text{-}375}$, the fitted k_{off} for MantADP release is 1.3 s^{-1} in BRB20 and 1.5 s^{-1} in BRB80, with $K_{0.5,MT}$ = 29 µM and 11 µM, respectively. For $Kif15_{1\text{-}375}$, the k_{off} is 0.8 s^{-1} in BRB20, with $K_{0.5,MT}$ = 7µM. The control was carried out with unpolymerised tubulin (■) in the presence of $Kif15_{19\text{-}375}$.
